# Genomic Evaluation of Multidrug-Resistant Extended-Spectrum β-Lactamase (ESBL)-Producing *Escherichia coli* from Irrigation Water and Fresh Produce in South Africa: A Cross-Sectional Analysis

**DOI:** 10.1021/acs.est.4c02431

**Published:** 2024-08-05

**Authors:** Loandi Richter, Stacey Duvenage, Erika Margarete du Plessis, Thabang Msimango, Manana Dlangalala, Muneiwa Tshidino Mathavha, Tintswalo Molelekoa, Degracious Moloko Kgoale, Lise Korsten

**Affiliations:** † Department of Plant and Soil Sciences, University of Pretoria, Hatfield, Pretoria 0001, South Africa; ‡ Department of Science and Innovation, National Research Foundation Centre of Excellence in Food Security, Bellville 7535, South Africa; § Food and Markets Department, Natural Resources Institute, University of Greenwich, Chatham ME4 4TB, United Kingdom

**Keywords:** one health, antimicrobial resistance, AMR, whole genome sequencing, WGS, food safety, environmental surveillance, ExPEC

## Abstract

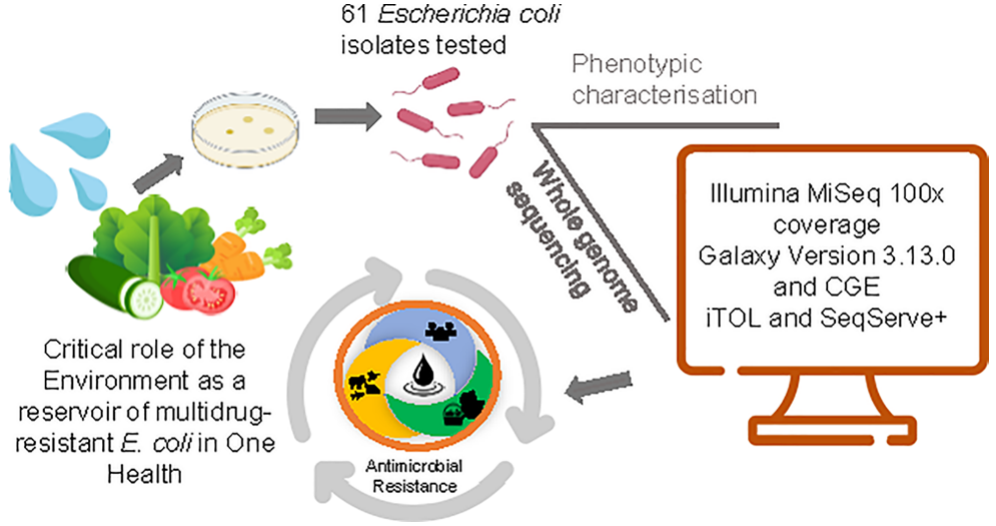

*Escherichia coli*, both commensal and pathogenic, can colonize plants and persist in various environments. It indicates fecal contamination in water and food and serves as a marker of antimicrobial resistance. In this context, 61 extended-spectrum β-lactamase (ESBL)-producing *E. coli* from irrigation water and fresh produce from previous studies were characterized using whole genome sequencing (Illumina MiSeq). The Center for Genomic Epidemiology and Galaxy platforms were used to determine antimicrobial resistance genes, virulence genes, plasmid typing, mobile genetic elements, multilocus sequence typing (MLST), and pathogenicity prediction. In total, 19 known MLST groups were detected among the 61 isolates. Phylogroup B1 (ST58) and Phylogroup E (ST9583) were the most common sequence types. The six ST10 (serotype O101:H9) isolates carried the most resistance genes, spanning eight antibiotic classes. Overall, 95.1% of the isolates carried resistance genes from three or more classes. The *bla*_CTX-M-1_, *bla*_CTX-M-14_, and *bla*_CTX-M-15_ ESBL genes were associated with mobile genetic elements, and all of the *E. coli* isolates showed a >90% predicted probability of being a human pathogen. This study provided novel genomic information on environmental multidrug-resistant ESBL-producing *E. coli* from fresh produce and irrigation water, highlighting the environment as a reservoir for multidrug-resistant strains and emphasizing the need for ongoing pathogen surveillance within a One Health context.

## Abstract 2

Limited research exists on the molecular characterization of environmental *Escherichia coli* in South Africa. This study reports the presence of virulence and antimicrobial resistance genes circulating in the water-plant-food nexus with implications for human health.

## Introduction

*Escherichia coli*, a gram-negative bacteria, is one of the most intensively studied microorganisms.^1^ As a commensal organism, it is among the first colonizing bacteria in the gastrointestinal tracts of humans and animals naturally occurring in the environment (water, soil, plants).^2,3^ Additionally, at least 11 pathotypes causing disease in humans and animals have been described and are classified into two categories: intestinal pathogenic (IPEC) and extraintestinal pathogenic (ExPEC) *E. coli*.^4,5^ The pathotype differentiation is based on the presence of specific virulence factors, mechanisms of infection, and interactions with host cells.^5^ Furthermore, *E. coli* strains belong to different phylogenetic groups, which are intertwined with virulence factors and the genetic substructures associated with different phylogeny, phenotypic, and genotypic traits.^6,7^ The most recent phylogenetic grouping of *E. coli* describes eight phylogroups (A, B1, B2, C, D, E, F, and G) based on the presence or absence of four genes (*ChuA*, *yjaA*, *TspE4.C2*, and *arpA*), with specific lifestyles and/or hosts attributed to each.^8−10^ Typically, *E. coli* infections among humans are associated with phylogroups B2 and, to a lesser extent, D, while phylogroups A and B1 are often associated with commensal *E. coli*.^8,11^

The IPEC pathotypes causing disease in humans and animals include enteropathogenic *E. coli* (EPEC), enterohemorrhagic/Shiga toxin-producing *E. coli* (EHEC/STEC), enterotoxigenic *E. coli* (ETEC), enteroaggregative *E. coli* (EAEC), diffusely adherent *E. coli* (DAEC), enteroinvasive *E. coli* (EIEC), and adherent invasive *E. coli* (AIEC).^12^ Generally, foodborne disease outbreaks have been associated with IPEC pathotypes, particularly EHEC/STEC.^13^ The characteristic virulence factors responsible for associated clinical symptoms of IPEC easily distinguish them from commensal *E. coli*; however, distinguishing ExPEC is difficult.^14^ Variants within the ExPEC group are classified according to the host and site of infection as uropathogenic *E. coli* (UPEC), neonatal meningitis *E. coli* (NMEC), avian pathogenic *E. coli* (APEC), and septicemia-associated *E. coli* (SEPEC).^11,12^ The virulence factors of ExPEC may be present in various combinations and can be divided into five main categories, namely, iron-sequestering systems (*iucD*, *irp2*, and *chuA*), adhesins (*papC*, *F10papA*, *sfaDE*, *afaBC III*, *iha*, *fimH*, *clpG*, *tsh*, and *hra*), invasin (*ibe10*), protectins (*TraT*, *OmpA*, and the capsular antigen K), and toxins (*ompT*, *ehxA*, *espP*, *hlyA*, *hlyD*, *vat*, *sat*, and *cnf 1*).^12,13^ ExPEC infections are being recognized as an emerging serious public health threat due to the increased acquisition of new and troubling antibiotic-resistance genes, leading to ineffective treatment options.^13^*E. coli* is globally reported as one of the leading pathogens responsible for human deaths associated with antimicrobial resistance.^15,16^

As *E. coli*, both commensal and pathogenic, can colonize and persist in various niches, it is often used as an indicator of fecal contamination in water and food safety system monitoring.^17^ More recently, it is also an indicator of antimicrobial resistance dynamics in a One Health context due to its genomic plasticity and frequent exposure to antimicrobial pressure.^18,19^ Indeed, the One Health paradigm implies that human and animal health and the environment are interdependent.^18^ Potential reservoirs for ExPEC include nonhuman reservoirs such as surface water, food animals, fresh produce, soil, sewage, and wastewater effluent.^15,20^ The ubiquity of *E. coli* renders it a One Health problem involving the water-plant-animal-food-public health interface; therefore, standardizing surveillance methodology across all reservoirs becomes important to be able to produce reliable, comparable data of the circulating genomic background.^21^

Whole genome sequencing (WGS) has become the tool of choice in laboratory-based outbreak investigations, particularly in public health.^21,22^ In addition to public health surveillance, within a food safety context, many high-income countries have successfully adopted WGS in routine food surveillance/monitoring systems.^21,23^ Higher accuracy insight into isolate relationships is provided with WGS analysis, making it possible to track trends associated with pathogen virulence and antimicrobial resistance. This can support risk assessment when combined with available metadata across all One Health domains.^21^ However, Richter et al.^24^ recently reported that the use of WGS in environmental surveillance studies in low-and middle-income countries (LMICs) remains low.

It is well documented that potential microbial contamination arises along many points throughout fresh produce production and supply systems.^25^ In South Africa, the dualistic fresh produce production system consists of highly regulated formal systems with commercial farms as well as the informal system, where predominantly small-scale farmers often have limited resources and infrastructure.^26^ However, across all fresh produce production in South Africa (both formal and informal), agricultural irrigation water sources predominantly include surface water (rivers, streams, dams, and canals) as well as borehole water.^28−30^ Typically, water used for irrigation will either be directly applied to the field from the specific water source or pumped into a holding dam or water reservoir until use.^30,31^ The current study aimed to evaluate the circulating antimicrobial resistance genes, virulence factors, and serotypes of 61 historically isolated multidrug-resistant ESBL-producing *E. coli* (2016–2019) from water and fresh produce samples in South Africa,^26−33^ using WGS. Furthermore, to establish baseline genomic information on the predicted pathogenicity of environmental isolates comparable to existing clinical data.

## Materials and Methods

### Multidrug-Resistant ESBL-Producing *E. coli* Selected for Whole Genome Sequence Analysis

Sequences of 61 multidrug-resistant ESBL-producing environmental *E. coli* isolates were retrieved from the National Center for Biotechnology Information (NCBI) GenBank database under the BioProject accession number PRJNA642017 for in-depth genomic characterization (Table 1). The de novo assembly metrics of all sequences are shown in the Supporting InformationTable 1. The contigs were subsequently submitted to the Galaxy platform (https://usegalaxy.eu/), Center for Genomic Epidemiology (CGE) platform (https://cge.cbs.dtu.dk/services/), and Technical University of Denmark (DTU) for bioinformatics analysis.

**Table 1:** Summary of 61 Environmental *E. coli* Strains Previously Reported in South African Point-Prevalence Studies for which the Short-Read Sequences were Retrieved from the National Center for Biotechnology Information (NCBI) Database for Metadata Whole Genome Sequence Analysis

isolate ID code	accession number	isolation source	reported phenotypic antibiotic resistance profile
**UPMP 588**	SAMN19374594	water	A10C-AP10C–CPM-30C-GM10C-CTX30C-CAZ30C–CPD10C–C10C-NE10C
**UPMP 589**	SAMN19374573	water	A10C-AP10C–CPM-30C-GM10C-CTX30C-CAZ30C–CPD10C–C10C-NE10C
**UPMP 615**	SAMN19374548	water	A10C-AP10C-TS25C-FOX30C–CPM30C-AUG30C-NE10C
**UPMP 1773**	SAMN16339888	water	A10C-AP10C-TS25C–CPM30C-T30C–C30C-CTX30C-CAZ30C–CPD10C-NE10C
**UPMP 1774**	SAMN16339889	water	A10C-AP10C-TS25C–CPM30C-T30C-T30C-GM10C-CTX30C-CAZ30C–CPD10C-AUG30C-NE10C
**UPMP 1995**	SAMN24818876	water	A10C-AP10C-TS25C-T30C–C30C-CTX30C–CPD10C-NE10C
**UPMP 1996**	SAMN24818877	water	A10C-AP10C-TS25C-T30C-CTX30C-CAZ30C–CPD10C-AUG30C-NE10C
**UPMP 1997**	SAMN19374561	water	A10C-AP10C-TS25C-T30C-CTX30C-CAZ30C–CPD10C-NE10C
**UPMP 2004**	SAMN19374584	water	A10C-AP10C-CTX30C-CAZ30C–CPD10C-AUG30C-NE10C
**UPMP 2005**	SAMN24818878	water	A10C-AP10C-FOX30C–C30C-CTX30C-CAZ30C–CPD10C-AUG30C-NE10C
**UPMP 2006**	SAMN24818879	water	A10C-AP10C-TS25C-T30C–C30C-CTX30C-CAZ30C–CPD10C-NE10C
**UPMP 2007**	SAMN19374572	water	A10C-AP10C-TS25C-FOX30C–C30C-CTX30C-CAZ30C–CPD10C-NE10C
**UPMP 2010**	SAMN19374579	water	A10C-AP10C-TS25C-FOX30C-T30C–C30C-CTX30C-CAZ30C–CPD10C-NE10C
**UPMP 2045**	SAMN19374575	water	not tested for phenotypic resistance
**UPMP 2062**	SAMN19374562	water	not tested for phenotypic resistance
**UPMP 2066**	SAMN24818881	water	not tested for phenotypic resistance
**UPMP 2087**	SAMN24818882	water	not tested for phenotypic resistance
**UPMP 2097**	SAMN24818883	water	not tested for phenotypic resistance
**UPMP 1722**	SAMN24818887	water	A10C-AP10C-TS25C–CPM30C-T30C-GM10C-CTX30C-CAZ30C–CPD10C-AUG30C-NE10C
**UPMP 1725**	SAMN24818888	water	A10C-AP10C-TS25C–CPM30C-T30C-CTX30C-CAZ30C–CPD10C-AUG30C-NE10C
**UPMP 1727**	SAMN19374590	water	A10C-AP10C-TS25C–CPM30C-T30C-CTX30C-CAZ30C–CPD10C-AUG30C-NE10C
**UPMP 1745**	SAMN24818908	water	A10C-AP10C-TS25C–CPM30C-T30C-CTX30C-CAZ30C–CPD10C-AUG30C-NE10C
**UPMP 1749**	SAMN19374589	water	A10C-AP10C-TS25C–CPM30C-T30C-CTX30C-CAZ30C–CPD10C-AUG30C-NE10C
**UPMP 1761**	SAMN19374598	water	A10C-AP10C-TS25C–CPM30C-IMI10C-T30C-GM10C–C30C-CTX30C-CAZ30C–CPD10C-AUG30C-NE10C
**UPMP 1772**	SAMN24818890	water	A10C-AP10C-TS25C–CPM30C-T30C–C30C-CTX30C-CAZ30C–CPD10C-AUG30C-NE10C
**UPMP 1785**	SAMN16339893	water	A10C-AP10C-TS25C–CPM30C-T30C-GM10C–C30C-CTX30C-CAZ30C–CPD10C-AUG30C-NE10C
**UPMP 1787**	SAMN24818891	water	A10C-AP10C-TS25C–CPM30C-T30C–C30C-CTX30C-CAZ30C–CPD10C-AUG30C-NE10C
**UPMP 1797**	SAMN24818892	water	A10C-AP10C-TS25C–CPM30C-T30C–C30C-CTX30C-CAZ30C–CPD10C-AUG30C-NE10C
**UPMP 1798**	SAMN16339898	water	A10C-AP10C-TS25C–CPM30C-T30C-GM10C–C30C-CTX30C-CAZ30C–CPD10C-AUG30C-NE10C
**UPMP 2117**	SAMN15421725	water	A10C-AP10C-FOX30C–CPM30C-T30C-GM10C-CTX30C-CAZ30C–CPD10C-AUG30C–C10C-N310C
**UPMP 2130**	SAMN15421738	water	A10C-AP10C-TS25C–CPM30C-IMI10C-T30C–C10C-NE10C
**UPMP 609**	SAMN19374555	fresh produce	A10C-AP10C-T30C-GM10C-AUG30C-NE10C
**UPMP 720**	SAMN19374549	fresh produce	A10C-AP10C–CPM10C-CTX30C-CAZ30C–CPD10C-AUG30C–C10C
**UPMP 723**	SAMN24818884	fresh produce	A10C-AP10C–CPM30C-CTX30C-CAZ30C–CPD10C-AUG30C–C10C-NE10C
**UPMP 767**	SAMN24818885	fresh produce	A10C-AP10C-TS25C–CPM30C-CTX30C-CAZ30C–CPD10C-AUG30C–C10C-NE10C
**UPMP 768**	SAMN19374597	fresh produce	A10C-AP10C-TS25C–CPM30C-T30C-CTX30C-CAZ30C–CPD10C-AUG30C-NE10C
**UPMP 784**	SAMN19374592	fresh produce	A10C-AP10C-TS25C–CPM30C-T30C-CTX30C-CAZ30C–CPD10C-AUG30C–C10C-NE10C
**UPMP 788**	SAMN19374554	fresh produce	A10C-AP10C-TS25C–CPM30C-T30C-GM10C-CTX30C-CAZ30C–CPD10C–C10CNE10C
**UPMP 790**	SAMN24818886	fresh produce	A10C-AP10C-FOX30C–CPM30C-IMI10C-T30C-GM10C-CTX30C-CAZ30C–CPD10C–C10C-NE10C
**UPMP 809**	SAMN19374564	fresh produce	A10C-AP10C-FOX30C–CPM30C-CTX30C-CAZ30C–CPD10C-AUG30C–C10C-NE10C
**UPMP 812**	SAMN19374550	fresh produce	A10C-AP10C-TS25C-FOX30C–CPM30C-IMI10C-T30C-GM10C-CTX30C–CPD10C-AUG30C–C10C-NE10C
**UPMP 818**	SAMN19374567	fresh produce	A10C-AP10C-FOX30C–CPM30C-IMI10C-T30C-CTX30C-CAZ30C–CPD10C-AUG30C–C10C-NE10C
**UPMP 819**	SAMN19374565	fresh produce	A10C-AP10C-TS25C-FOX30C–CPM30C-T30C-CTX30C-CAZ30C–CPD10C-AUG30C–C10C-N310C
**UPMP 1126**	SAMN19374556	fresh produce	A10C-AP10C-TS25C-FOX30C–CPM30C-T30C-GM10C–C30C–CIP5C–S10C-NA30C-CTX30C-CAZ30C–CPD10C-AUG30C-NE10C-KF30C–N300C
**UPMP 1129**	SAMN19374544	fresh produce	A10C-AP10C-TS25C-FOX30C–CPM30C-T30C-GM10C–C30C–CIP5C–S10C-NA30C-CTX30C-CAZ30C–CPD10C-AUG30C-NE10C-KF30C–N300C
**UPMP 1131**	SAMN19374545	fresh produce	A10C-AP10C-TS25C-FOX30C–CPM30C-T30C-GM10C–CIP5C–S10C-NA30C-CTX30C-CAZ30C–CPD10C-AUG30C-NE10C-KF30C–N300C
**UPMP 1515**	SAMN19374559	fresh produce	T30C-GM10C–CIP5C-NA30C-KF30C
**UPMP 1524**	SAMN24818870	fresh produce	A10C-AP10C-TS25C-T30C-GM10C–C30C–CIP5C–S10C-N3C-CTX30C-KF30C–N300C
**UPMP 1531**	SAMN19374553	fresh produce	A10C-AP10C-TS25C-T30C–C30C–CIP5C–S10C-NA30C-CTX30C-KF30C–N300C
**UPMP 1542**	SAMN24818872	fresh produce	A10C-AP10C-TS25C-T30C–C30C–CIP5C–S10C-NA30C-CTX30C-KF30C–N300C
**UPMP 1545**	SAMN24818873	fresh produce	A10C-AP10C-TS25C-T30C–C30C–CIP5C-NA30C-CTX30C-KF30C–N300C
**UPMP 1547**	SAMN24818874	fresh produce	A10C-AP10C-TS25C-T30C–C30C–CIP5C–S10C-NA30C-CTX30C-KF30C–N300C
**UPMP 1548**	SAMN19374576	fresh produce	A10C-AP10C-TS25C-T30C-GM10C–C30C-S10C-NA30C-CTX30C-KF30C–N300C
**UPMP 1549**	SAMN24818871	fresh produce	A10C-AP10C-TS25C-T30C-GM10C–C30C-S10C-NA30C-CTX30C-KF30C–N300C
**UPMP 1551**	SAMN24818875	fresh produce	A10C-AP10C-TS25C-T30C–C30C-S10C-NA30C-CTX30C-KF30C–N300C
**UPMP 1716**	SAMN15905474	fresh produce	A10C-AP10C–CPM30C-IMI10C-T30C-CTX30C-CAZ30C–CPD10C-AUG30C-NE10C
**UPMP 1991**	SAMN19374581	fresh produce	A10C-AP10C-TS25C-T30C–C30C-CTX30C-CAZ30C–CPD10C-NE10C
**UPMP 1993**	SAMN19374586	fresh produce	A10C-AP10C-FOX30C-CTX30C-CAZ30C–CPD10C-NE10C
**UPMP 2011**	SAMN24818880	fresh produce	A10C-AP10C-T30C–C30C-CTX30C-CAZ30C–CPD10C-AUG30C-NE10C
**UPMP 2050**	SAMN19374582	fresh produce	not tested for phenotypic resistance
**UPMP 2120**	SAMN15421728	fresh produce	A10C-AP10C-TS25C-FOX30C–CPM30C-IMI10C-CTX30C-CAZ30C–CPD10C–C10C-NE10C

### Phylogenetic Screening

All genomes were annotated using Prokka (Galaxy Version 1.14.6 + galaxy1),^34^ and the *E. coli* core genome alignments were constructed using Roary (Galaxy Version 3.13.0 + galaxy2)^35^ based on the genome annotation files (gff3 file). The default parameters (95% identity for blastp and 99% of isolates a gene must be in to be core) were used in Roary to classify the core/unique genes. Subsequently, the “core gene alignment” Roary results were used to construct a phylogenetic tree using Fasttree (Galaxy Version 2.1.10 + galaxy1) and visualized using iTOL.^36^ A core genome MLST (cgMLST) analysis was additionally performed with the default settings using cgMLSTFinder-1.2 on the CGE platform^37,38^ and visualized in iTOL. The minimum spanning tree from the *E. coli* isolates based on the MLST scheme was generated using SeqSphere+.^39^ The different phylogroups of the *E. coli* isolates were determined using *in silico* ClermonTyping.^9^

### Gene Screening

Using the CGE platform (https://cge.cbs.dtu.dk/services/), the sequence types, serotypes based on lipopolysaccharide (O-antigen) and capsular flagella (protein; H-antigen), and virulence genes were determined with Multilocus Sequence Typing (MLST; version 2.2), SeroTypeFinder (version 2.0), and VirulenceFinder (version 2.0), respectively.^40−42^ Default parameters were considered for all of the software used unless otherwise indicated. With ABRicate (https://github.com/tseemann/abricate), the AMR gene presence was corroborated using the Comprehensive Antibiotic Resistance Database (CARD), ARG-ANNOT, ResFinder, NCBI AMRFinder Plus, and MEGA Res databases,^43−48^ while the presence of metal resistance genes was determined with BacMet version 2.0.^49^ Furthermore, mobile genetic elements and their association with virulence and antimicrobial resistance genes were determined with MobileElementFinder (Version 1.0.3),^50^ and the presence of integrons with IntegronFinder version 2.0,^51^ while PathogenFinder version 1.1 was used to predict the pathogenicity of the *E. coli* isolates toward human hosts.^52^

## Results

### Phylogroups, Sequence Types, and Serotypes of the *E. coli* Isolates

The phylogenetic grouping showed that *E. coli* belonging to phylogroups A, B1, C, D, E, F, and G were recovered from water samples, and *E. coli* that belonged to phylogroups A, B1, B2, D, E, and G were recovered from fresh produce samples. Of the 61 *E. coli* isolates, phylogroups A (31.15%) and B1 (27.87%) were the most common in the environmental samples. The B2 isolates (3.28%) were recovered from fresh produce samples only, while isolates belonging to phylogroups D (6.56%) and E (14.75%) were recovered from water and fresh produce samples. Interestingly, four isolates (6.56%) from both water and fresh produce samples belonged to phylogroup G, which is closely related to phylogroup B2.^10^

**Figure 1:**
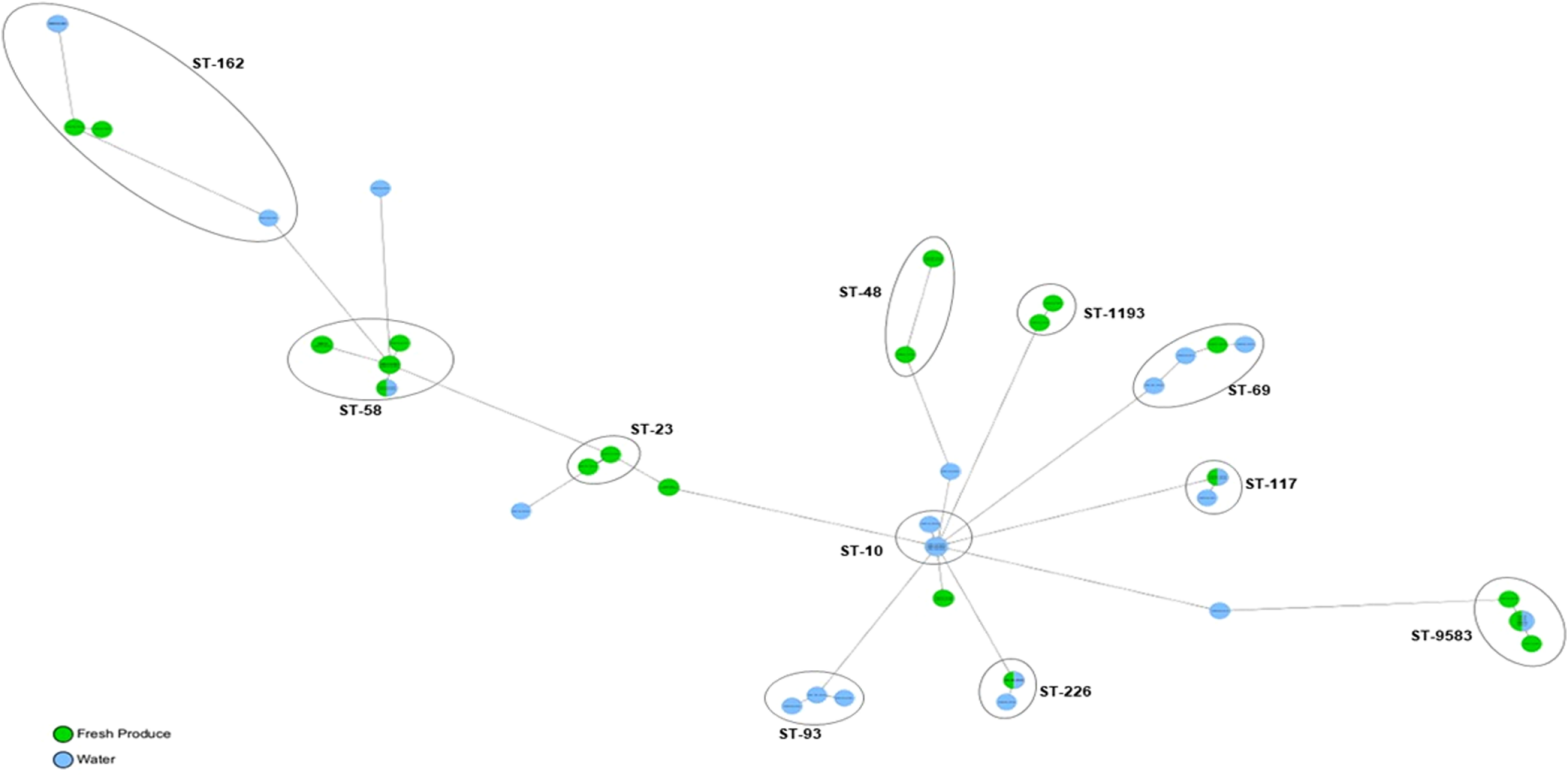
cgMLST-based minimum spanning tree of 61 *E. coli* isolates recovered from water and fresh produce from formal and informal fresh produce production systems in South Africa. Isolates belonging to the same dominant sequence types (ST) are circled and labeled, and the isolation source is shown in different colors.

**Figure 2:**
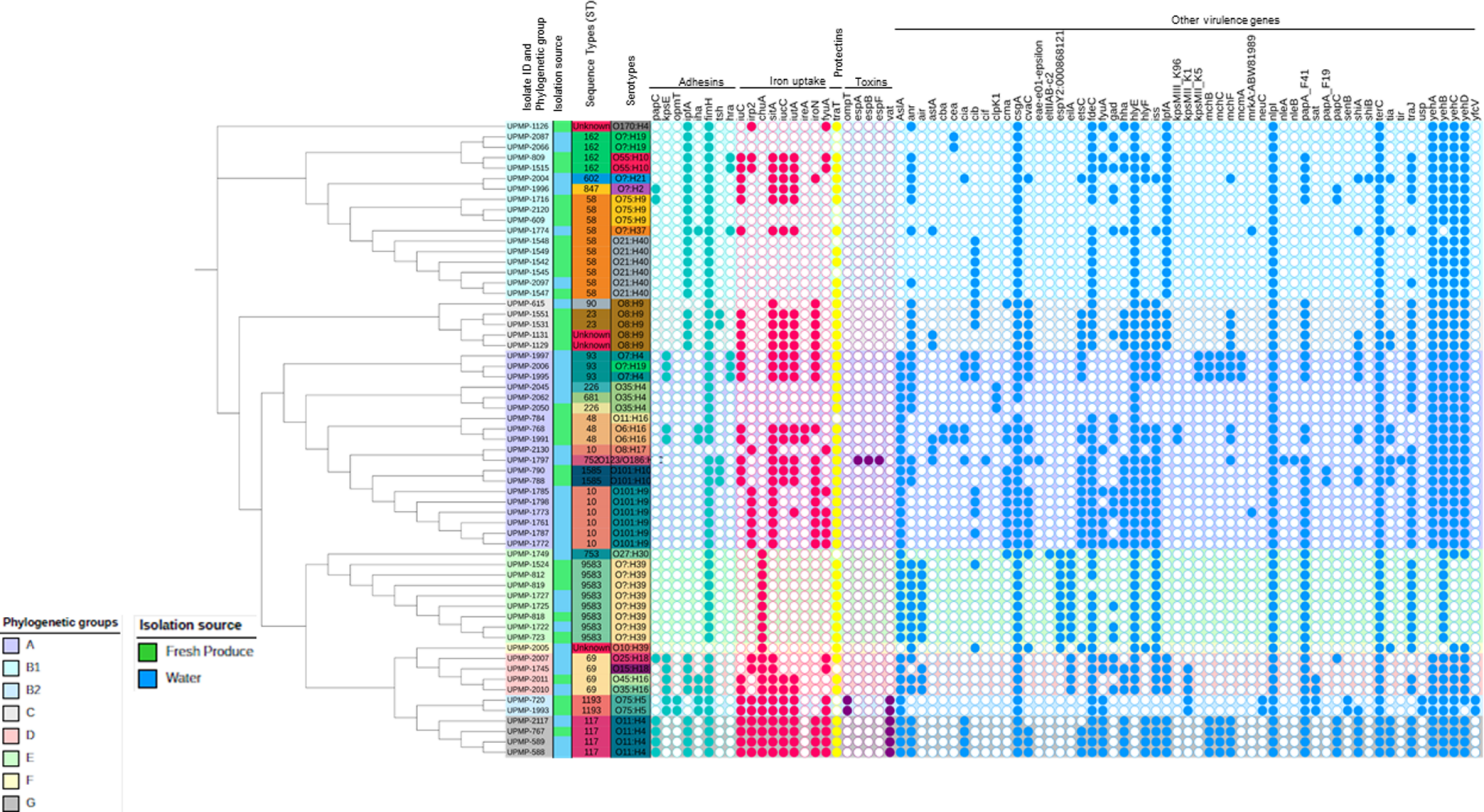
cgMLST-based phylogenetic tree showing the distribution of virulence genes by the phylogroup, sequence type (ST), serogroup, and isolation source (water, blue and fresh produce, green) of *E. coli*. The colored circles indicate the presence (filled) or absence (open circle) of the different virulence genes with Adhesins, Iron uptake genes, Protectins, and Toxins typically associated with ExPEC.

A total of 19 known MLST groups were detected among the 61 isolates (Figure 1 and Tables 3–5). ST58 (*n* = 10, 16.39%), belonging to phylogroup B1, and ST9583 (*n* = 8, 13.11%), belonging to phylogroup E, were the most common *E. coli* sequence types associated with the environmental (water and fresh produce) samples. Within phylogroup B1 isolates, other STs found included ST162, ST602, and ST847. The ST10 (*n* = 6, 11.48%) isolates were restricted to the water samples and detected only in isolates belonging to phylogroup A (Figure 1). Other STs associated with phylogroup A were ST48, ST93, ST226, ST681, ST752, and ST1585. ST1193 was detected in phylogroup B2 and ST117 in phylogroup G. Four isolates (6.56%) within phylogroups B1, E, and F belonged to unknown STs, while most of the other MLST groups were detected to a limited extent (Figure 2). Using Enterobase,^53^ the unknown STs were identified within the MLST-Enterobase (ST210d7d18a802c59df81880a978149a02c49a6021b, STc75778699e0a2b1faca8b5d6f9051eb7d9defca4, and ST85e7b10eb1371e1fae7d8bf12c0066e6a995add0) and MLST-Pasteur (STb0618816d6163930f5c1952a39b99044904119f5, ST7829ecb9c01fd0f5134a9452e5ded95cdbc670dc, and STcddffc6167c10ddd07704ddd888c485cedb717d2) databases.

No distinct pattern was observed among the MLST groups, phylogroups, and serotypes (i.e., O- and H-typing). A total of 14 (22.95%) isolates had an untypeable O-type, while the H-types for these isolates varied between H19, H2, H21, H37, and H39. One isolate from fresh produce belonging to phylogroup A was determined to contain two O-types on the same contig (Figure 2). Eight isolates (ST10, ST753, or ST1585) with the O101 serogroups had either H9 or H10 types and belonged predominantly to phylogroup A (Figure 2). In total, a diversity of 27 serotypes were detected across the complete collection (Figure 2).

### Characteristics of Virulence Factors among the *E. coli* Isolates

The virulence genes detected belonged to the adhesins, nutritional/metabolic, biofilm, invasion, and effector delivery systems virulence factor categories.^54^ The distribution of the different virulence genes mostly depended on the phylogroups and sequence types (Figure 2). No distinct difference was seen in virulence-associated genes detected in *E. coli* isolated from water compared to those isolated from fresh produce samples. The most frequent virulence genes identified within the adhesin category were the UPEC-associated *fimH*, followed by the APEC-associated *ipfA*. Within the nutritional/metabolic category, the iron uptake virulence genes were predominant in isolates that belonged to phylogroup G (Figure 2). Furthermore, the *chuA* virulence factor was the only gene that regulated iron uptake detected in isolates belonging to phylogroup E. The UPEC-associated *traT* protectin virulence factor was present in 81.97% (*n* = 50) of the isolates. Except for one phylogroup A isolate where *espA*, *espB*, and *espF* were found, the toxin category genes (*ompT*, *n* = 2 and *vat*, *n* = 4) were exclusively present in isolates from phylogroups B2 and G, respectively (Figure 2).

### Antimicrobial Resistance Genes

**Figure 3:**
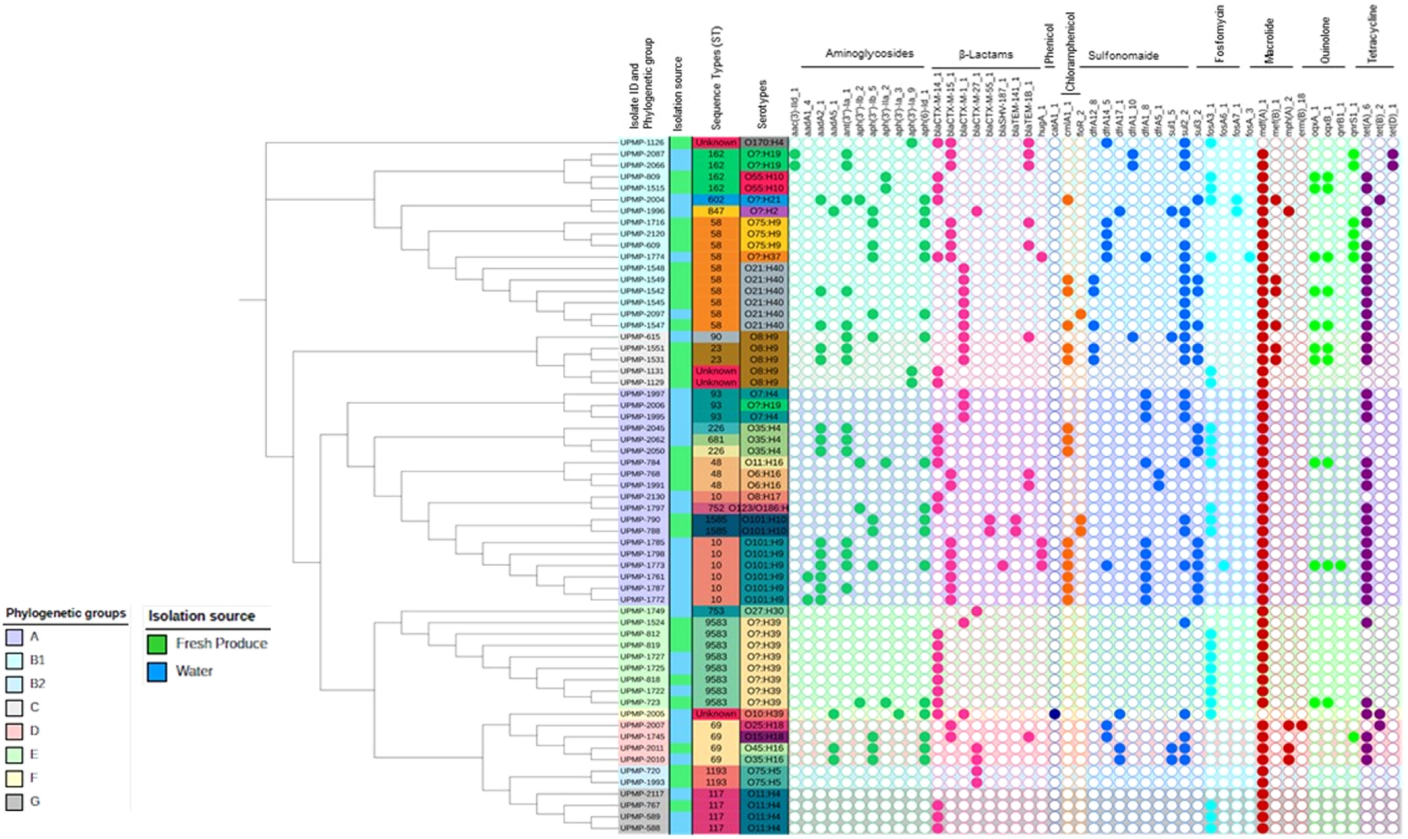
cgMLST-based phylogenetic tree showing the distribution of antimicrobial resistance genes by the phylogroup, sequence type (ST), serogroup, and isolation source (water: blue and fresh produce: green) of *E. coli*. The colored circles indicate the presence (filled) or absence (open circle) of the different genes within the different antibiotic classes.

Only 95.1% of the 61 isolates presented a potentially multidrug-resistant genotype with resistant genes in three or more antibiotic classes present (Figure 3). Overall, 60.66% of the *E. coli* isolates had aminoglycoside resistance genes present. The most common aminoglycoside-modifying enzyme encoding genes were *aph(6)-Id* followed by *ant(3*″*)-Ia, aadA2*, and *aph(3*″*)-Ib*. The phenotypic antimicrobial resistance patterns of the *E. coli* isolates from the individual cross-sectional studies^26,27,30−33^ showed that at least 55 of the *E. coli* isolates exhibited a phenotypic multidrug resistance profile, with resistance to different antibiotics in at least three different antibiotic classes (Table 2).

**Table 2:** Antimicrobial Resistance Results of *E. coli* Isolates from Water and Fresh Produce Samples in Formal and Informal Production Systems in South Africa

		number (%) of resistant isolates	
antibiotic class	antibiotic	fresh produce (*n* = 29)	water (*n* = 26)
penicillin	ampicillin	28 (97)	26 (100)
	amoxicillin	28 (97)	25 (96)
	augmentin	14 (48)	19 (73)
sulfonamide	trimethoprim-sulfamethoxazole	19 (66)	20 (77)
carbapenem	imipenem	5 (17)	3 (12)
tetracycline	tetracycline	22 (76)	21 (81)
aminoglycoside	neomycin	19 (66)	25 (96)
	gentamicin	12 (41)	7 (27)
	nitrofurantoin	11 (38)	
	streptomycin	10 (34)	
chloramphenicol	chloramphenicol	24 (83)	15 (58)
quinolone	nalidixic acid	12 (41)	
fluoroquinolones	ciprofloxacin	11 (38)	
cephalosporin	cephalothin	12 (41)	
	cefoxitin	10 (34)	5 (19)
	cefpodoxime	18 (62)	23 (88)
	cefotaxime	26 (90)	24 (92)
	ceftazidime	19 (66)	22 (85)
	cefepime	17 (59)	18 (69)

The dominant ESBL-encoding genes in the confirmed ESBL-producing *E. coli* were *bla*_CTX-M-14_ (*n* = 24, 39, 34%), *bla*_CTX-M-15_ (*n* = 17, 27, 87%), and *bla*_CTX-M-1_ (*n* = 14; 22, 95%), found in isolates belonging to all of the different phylogenetic groups (Figure 3). Other β-lactamase genes present predominantly in phylogroups A, B1, and C include *bla*_CTX-M-55_, *bla*_SHV-187_, *bla*_TEM-1B_, *bla*_TEM-141_, and *hugA*. Additionally, in selected isolates belonging to phylogroups A-B2, the *bla*_CTX-M-27_ ESBL gene was present (Figure 3). Overall, a higher percentage of water *E. coli* isolates showed resistance against third-generation cephalosporins (cefpodoxime, cefotaxime, ceftazidime, and cefepime) than *E. coli* isolates from fresh produce (Table 1). Although the phylogroup G isolates harbored fewer resistance genes compared to isolates from other phylogroups (Figure 3), the WGS-predicted phenotype and phenotypic antimicrobial resistance profiles presented as multidrug-resistant. The phylogroup B2 isolates harbored β-lactam and macrolide resistance genes; however, the phenotypic profiles only showed expression of β-lactamase enzymes. Interestingly, the one phylogroup F isolate was the only one to harbor the *catA1* resistance gene. The chloramphenicol exporter resistance genes (*cmlA1* and *floR*) were identified in 24.6% (*n* = 15) and 4.9% (*n* = 3) of the isolates, respectively, all belonging to phylogroups A (*n* = 9 *cmlA*, *n* = 2 *floR*), B1 (*n* = 4 *cmlA*, *n* = 1 *floR*), and C (*n* = 2 *cmlA*). Out of the 55 isolates tested for antimicrobial resistance susceptibility, 71% were resistant against chloramphenicol (Table 2). Within the sulfonamide class, the *sul2* resistance gene was mostly present (28/61), followed by *sul3* (15/61) (Figure 3). Additionally, the *dfrA1* gene encoding trimethoprim resistance was present (11/61), and phenotypic resistance against trimethoprim-sulfamethoxazole was observed in 39 of the 55 isolates (Table 2). Only 27 isolates harbored fosfomycin resistance genes, with *fosA3* present in 25 of these isolates. Of all of the isolates, the six ST10 (serotype O101:H9) isolates carried the most resistance genes, with genes from eight different antibiotic classes present (Figure 3). These isolates all had similar multidrug-resistant phenotypes with resistance against antibiotics within the penicillin, sulfonamide, tetracycline, aminoglycoside, and cephalosporin antibiotic classes. Interestingly, no hits were found for predicted or experimentally confirmed metal resistance genes in any of the isolates.

### Mobile Genetic Elements

Mobile genetic elements associated with similar virulence and antimicrobial resistance genes were present in *E. coli* isolates from water and fresh produce samples (Table 3). Interestingly, only water sample *E. coli* isolates had Inc.FII and Inc.FII(pRSB107) plasmids associated with aminoglycoside, tetracycline, and chloramphenicol antimicrobial resistance genes (Table 4), while *E. coli* from water and fresh produce samples carried the same plasmids (Inc.FII and Inc.FII(pRSB107)) with associated virulence factors. The ESBL-encoding genes associated with mobile genetic elements were *bla*_CTX-M-1_ (associated with ISEc9 and Inc.1), *bla*_CTX-M-14_ (associated with IS26), *bla*_CTX-M-15_ (associated with ISEc9 and ISKpn19), and *bla*_CTX-27_ (associated with IS102) (Tables 3–5). In total, 31/61 isolates did not contain integrons, while three types of elements (complete integron, *In0*, and CALIN) were identified in the remaining isolates. IntegronFinder distinguishes a complete integron as an integron with an integron integrase nearby *attC* sites. From the current study, three isolates from leafy green vegetables and 12 isolates from river, borehole, or dam water contained complete Class 1 integrons. An *In0* element is distinguished as an integron integrase only, without any *attC* site nearby, and three isolates from cucumber, spinach, and canal water carried *In0* elements. CALIN elements are described as clusters of *attC* sites lacking integrase nearby or a degraded integron. In the current study, nine isolates from dam water, cabbage, spinach, and apple samples carried CALIN elements. Overall, all of the *E. coli* isolates showed a > 90% predicted probability of being a human pathogen. This follows as the PathogenFinder tool provides a fast estimation of the pathogenic potential of bacteria based on the identification of gene families that correlate with pathogenicity in known and unknown strains.^52^

**Table 3:** Mobile Genetic Elements Associated with Virulence and Antimicrobial Resistance Genes in *E. coli* (Grouped According to Sequence Type) Isolated from Water and Fresh Produce Samples in South Africa

			mobile genetic elements		genes associated with mobile genetic elements	
source	isolate ID code (UPMP-*)	accession number (SAMN*)	insertion sequence	plasmid	virulence	resistance
***E. coli* ST62**						
river water	*2066	*24818881	ISCfr1			*blaTEM-1B; aac(3)-Iid*
			MITEEc1		*nlpI; terC*	
			IS100		*yehD; yehA; yehC; yehB*	
			MITEKpn1		*fdeC*	
borehole water	*2087	*24818882	ISCfr1			*aac(3)-Iid; bla*_*TEM-1B*_
			IS100		*yehB; yehA; yehD; yehC*	
			MITEKpn1		*(gad) (fdeC)*	
			MITEEc1		*nlpI; terC*	
tomato	*809	*19374564		Inc.X1		*aph(3′)-IIa*
				Inc.FIB(AP001918)	*hlyF; ompT*	
			ISEc1		*(fdeC) (yehC; yehA; yehD; yehB)*	
			ISEc31		*hha*	
			MITEEc1		*(nlpI; terC) (terC; hra)*	
***E. coli* ST117**						
spinach	*767	*24818885		Inc.FII	*traJ; traT; traJ; anr*	
			ISEc38		*hha; fyuA; irp2*	
			IS30		*mchB; mchF; mchC; papC*	
			IS421		*ompT*	
water reservoir	*2117	*15421725		Inc.FII	*traJ; anr; traT; traJ*	
			ISEc38		*hha*	
			ISEc17		*mchF; mchC; mchB*	
water reservoir	*588	*19374594		Inc.FII	*traJ; traT; traJ; anr*	
			IS629		*shiA; tia; shiA*	
			IS30		*mchC; papC; mchF; mchB*	
			ISEc38		*hha; fyuA; irp2*	
			MITEEc1		*terC*	
			IS421		*ompT*	
water reservoir	*589	*19374573	IS629		*tia; shiA; shiA*	
			IS30		*mchB; papC; mchC; mchF*	
				Inc.FII	*traT; traJ; anr; traJ*	
			MITEEc1		*terC*	
			ISEc38		*fyuA; hha; irp2*	
			IS421		*ompT*	
***E. coli* ST266**						
borehole water	*2045	*19374575		Inc.FII(29)	*anr*	
chinensis	*2050	*19374582		Inc.FII(29)	*anr*	
			IS609		*fimH*	
***E. coli* ST69**						
canal water	*1745	*24818908	ISEc9			*bla*_*TEM-1B*_*; qnrS1; aph(6)-Id; aph(3*″*)-Ib; sul2; bla*_*CTX-M-15*_
			MITEEc1		*(terC) (yehC; yehA; yehD; yehB)*	
			ISEc46		*irp2; fyuA*	
			ISEc1		*ompT*	
river water	*2007	*19374572	ISEc9		*irp2; fyuA*	*bla*_*CTX-M-15*_
			IS6100			*dfrA14; mph(A)*
			IS5		*yehD; yehC; yehA; yehB*	
			ISKpn24		*terC*	
river water	*2010	*19374579	IS6100			*mph(A); qacE; dfrA17; sul1; aadA5*
			IS102			*bla*_*CTX-M-27*_
			MITEEc1		*yehA; yehD; yehC; yehB*	
			IS640		*papA_F43*	
			ISEc10		*kpsMII; kpsE*	
			ISEc1		*ompT*	
spinach	*2011	*24818880	IS6100			*qacE; dfrA17; mph(A); sul1; aadA5*
			IS102			*bla*_*CTX-M-27*_
			MITEEc1		*yehD; yehB; yehA; yehC*	
			ISEc10		*kpsE; kpsMII*	
			IS640		*papA_F43*	
***E. coli* ST9583**						
canal water	*1722	*24818887	MITEEc1		*terC*	
			IS609		*yehB*	
canal water	*1725	*24818888	MITEEc1		*terC*	
dam water	*1727	*19374590	MITEEc1		*terC*	
			IS609		*yehB*	
spinach	*723	*24818884		Inc.HI2; Inc.HI2A	*terC*	*aph(3*″*)-Ib; aph(6)-Id; aph(3*″*)-Ib*
			IS609		*yehB*	
			MITEEc1		*terC*	
spinach	*818	*19374567	MITEEc1		*terC*	
onions	*812	*19374550	IS26			*bla*_*CTX-M-14*_*; fosA3*
			MITEEc1		*terC*	
			IS609		*yehB*	
green beans	*819	*19374565	MITEEc1		*terC*	
tomato	*1524	*24818870		Inc.I1	*cib*	*tet(A); sul2*
			ISEc9			*bla*_*CTX-M-1*_
			MITEEc1		*terC*	
				Inc.FIB(AP001918)	*ompT*	
***E. coli* ST58**						
canal water	*1749	*19374589	IS102			*bla*_*CTX-M-27*_
borehole water	*2097	*24818883	IS26			*sul2*
			ISSbo1			*tet(A)*
			ISEc9			*bla*_*CTX-M-1*_
			ISEc60		*yehC; yehB; yehA; yehD*	
				Inc.FII(29)	*anr*	
dam water	*1774	*16339889	ISKpn19			*tet(A); qnrS1*
			ISKox3		*sitA; iucC; iutA*	*sitABCD*
				Inc.FII(pCoo)	*traJ; traT; anr*	
				Inc.B/O/K/Z	*traT*	
			IS100		*iha*	
			MITEEc1		*(yehD; yehC) (terC) (terC) (iss)*	
			IS30		*hha*	
apple	*1548	*19374576		Inc.I1	*cib*	*tet(A); sul2*
			ISEc9			*bla*_*CTX-M-1*_
			ISEc60		*yehB; yehA; yehD; yehC*	
			ISEc31		*terC*	
apple	*1549	*24818871		Inc.I1		*tet(A)*
			ISVsa3		*cib*	*sul2*
			ISEc9			*bla*_*CTX-M-1*_
			MITEEc1		*(terC) (fdeC)*	
cabbage	*1547	*24818874	ISSbo1			*tet(A)*
			ISVsa3			*sul2*
			ISEc9			*bla*_*CTX-M-1*_
			ISEc31		*terC*	
			MITEEc1		*(fdeC) (yehA; yehC; yehB; yehD)*	
carrots	*1545	*24818873		Inc.I1	*cib*	*tet(A); bla*_*CTX-M-1*_*; sul2*
			ISEc60		*yehD; yehC; yehA; yehB*	
			MITEEc1		*fdeC*	
			ISEc31		*terC*	
cucumber	*1716	*15905474	IS5075			*bla*_*TEM-1B*_*; aph(6)-Id; tet(A); aph(3*″*)-Ib; sul2*
			ISKpn19			*qnrS1; bla*_*CTX-M-15*_
			MITEEc1		*(terC) (nlpI)*	
spinach	*1542	*24818872	ISVsa3			*sul2*
				Inc.I1		*tet(A)*
			ISEc9			*bla*_*CTX-M-1*_
			ISEc60		*yehC; yehA; yehD; yehB*	
			ISEc1		*fdeC*	
			MITEEc1		*terC*	
spinach	*2120	*15421728	ISKpn19			*qnrS1; bla*_*CTX-M-15*_
			ISEc31		*terC*	
			IS629		*csgA; hlyE*	
			IS609		*gad*	
			MITEEc1		*nlpI*	
spinach	*609	*19374555	ISKpn19			*qnrS1; bla*_*CTX-M-15*_
			MITEEc1		*terC*	
			IS629		*csgA; hlyE*	

**Table 4:** Mobile Genetic Elements Associated with Virulence and Antimicrobial Resistance Genes in *E. coli* (Grouped According to the Sequence Type) Isolated from Water Samples only in South Africa

			mobile genetic elements		genes associated with mobile genetic elements	
source	isolate ID code (UPMP-*)	accession number (SAMN*)	insertion sequence	plasmid	virulence	resistance
***E. coli* ST10**						
dam water	*1761	*19374598	ISEc9		*irp2; fyuA*	*bla*_*CTX-M-15*_
				Inc.FIB(AP001918)	*hlyF; etsC; ompT*	
				Inc.FII(pRSB107)	*traT*	
			ISKox3		*hlyE*	
			IS30		*hha*	
	*1772	*24818890	ISEc9		*irp2; fyuA*	*bla*_*CTX-M-15*_
			ISKox3		*hlyE*	
				Inc.FIB(AP001918)	*hlyF; ompT; etsC*	
	*1773	*16339888		Inc.FII(pRSB107)	*traT*	*tet(A); aadA2b; cmlA1*
			Tn5403			*qnrB1*
			IS5075			*aph(6)-Id; aph(3*″*)-Ib; sul2*
			ISEc9			*bla*_*CTX-M-15*_
				Inc.FIB(AP001918)	*ompT; hlyF; etsC*	
				Inc.I1	*cib*	
			ISKox3		*hlyE*	
			IS30		*hha*	
	*1785	*16339893		Inc.FII(pRSB107)	*traT*	*aadA2b; tet(A); cmlA1*
			ISEc9		*irp2; fyuA*	*bla*_*CTX-M-15*_
				Inc.FIB(AP001918)	*etsC; ompT; hlyF*	
			ISKox3		*hlyE*	
			IS30		*hha*	
				Inc.I1	*cib*	
	*1787	*24818891	ISEc9		*fyuA; irp2*	*bla*_*CTX-M-15*_
			ISSbo1			*tet(A); aadA2b; cmlA1*
			ISKox3		*hlyE*	
				Inc.FIB(AP001918)	*hlyF; etsC; ompT*	
				Inc.FII(pRSB107)	*traT*	
				Inc.I1	*cib*	
			IS30		*hha*	
	*1798	*16339898		Inc.FII(pRSB107)	*traT*	*aadA2b; tet(A); cmlA1*
			ISEc9		*fyuA; irp2*	*bla*_*CTX-M-15*_
				Inc.FIB(AP001918)	*etsC; ompT; hlyF*	
				Inc.I1	*cib*	
			ISKox3		*hlyE*	
			IS30		*hha*	
	*2130	*15421738	ISEc9			*bla*_*CTX-M-14*_
			IS6100			*dfrA14*
				Inc.FII(pRSB107)	*anr*	
***E. coli* ST90**						
river water	*615	*19374548	ISVsa3		*cib*	*sul2*
				Inc.FII		*tet(A)*
			ISEc9			*bla*_*CTX-M-1*_
			MITEEc1		*yehD; yehA; yehB; yehC*	
			MITEEc1		*nlpI; terC; terC*	
				Inc.FIB(AP001918)	*ompT; hlyF*	
			MITEEc1		*fdeC*	
			MITEEc1		*iss*	
***E. coli* ST93**						
dam water	*1995	*24818876		Inc.I1		*tet(A)*
			ISEc9			*bla*_*CTX-M-1*_
			IS100		*mchB; kpsE; kpsMII_K5; mchC; mcmA; mchF*	
				Inc.FIB(AP001918)	*hlyF; ompT*	
			MITEEc1		*hra*	
				Inc.FII	*traT; anr; traJ; traJ*	
			ISEc31		*shiA*	
			ISEc1		*ompT*	
	*1997	*19374561		Inc.I1		*tet(A); bla*_*CTX-M-1*_
			IS100		*kpsE; mchC; kpsMII_K5; mchB; mcmA; mchF*	
				Inc.FIB(AP001918)	*hlyF; etsC; etsC; ompT*	
				Inc.FII	*traT; traJ; anr; traJ*	
			ISEc31		*shiA*	
			ISEc1		*ompT*	
	*2006	*24818879		Inc.I1		*tet(A)*
			ISEc9			*bla*_*CTX-M-1*_
			ISEc31		*shiA*	
				Inc.FIB(AP001918)	*etsC; ompT; hlyF; etsC*	
				Inc.FII	*traT; traJ; anr; traJ*	
			IS100		*mcmA; kpsE; kpsMII_K5; mchB; mchC; mchF*	
			ISEc1		*ompT*	
			MITEEc1		*hra*	
***E. coli* ST602**						
river water	*2004	*19374584		Inc.FII		*aph(3*″*)-Ib; aph(3*″*)-Ib; aph(3*″*)-Ib; aph(3*″*)-Ib; aph(6)-Id*
				Inc.FIB(AP001918)	*iroN; iss; etsC; ompT; etsC; hlyF*	
			MITEEc1		*fdeC*	
			ISKpn24		*cia; cvaC; mchF*	
***E. coli* ST681**						
river water	*2062	*19374562		Inc.FII(29)	*anr*	
			IS609		*fimH*	
***E. coli* ST752**						
dam water	*1797	*24818892		Inc.FIB(AP001918)	*ompT; hlyF*	
			ISEc1		*fdeC*	
			ISEic2		*astA*	
				Inc.FII(pSE11)	*anr*	
***E. coli* ST847**						
dam water	*1996	*24818877	IS6100			*dfrA17; qacE; mph(A); sul1; aadA5*
			IS102			*bla*_*CTX-M-27*_
			MITEEc1		*nlpI*	
			IS30		*papC*	
			MITEEc1		*yehD; yehA; yehB; yehC*	
			ISEc1		*ompT*	
			MITEEc1		*terC*	
***E. coli* ST85e7b10eb1371e1fae7d8bf12c0066e6a995add0**						
river water	*2005	*24818878	ISVsa3			*sul2*

**Table 5:** Mobile Genetic Elements Associated with Virulence and Antimicrobial Resistance Genes in *E. coli* (Grouped According to the Sequence Type) Isolated from Fresh Produce Samples only in South Africa

			mobile genetic elements		genes associated with mobile genetic elements	
source	isolate ID code (UPMP-*)	accession number (SAMN*)	insertion sequence	plasmid	virulence	resistance
***E. coli* ST23**						
cabbage	*1531	*19374553	ISVsa3			*sul2*
				Inc.I1		*tet(A)*
			ISEc9			*bla*_*CTX-M-1*_
			MITEEc1		*(yehC; yehA;yehB; yehD) (nlpI) (terC)*	
				Inc.FIB(AP001918)	*etsC; hlyF; etsC; ompT*	
apple	*1551	*24818875		Inc.I1		*tet(A); bla*_*CTX-M-1*_
			ISVsa3			*sul2*
			MITEEc1		*(yehB; yehA; yehD; yehC) (terC) (nlpI)*	
				Inc.FIB(AP001918)	*etsC; ompT; etsC; hlyF*	
			ISEc1		*ompT*	
***E. coli* ST48**						
lettuce	*1991	*19374581	ISEc9			*bla*_*CTX-M-15*_
			IS5		*tia*	
			IS629		*ireA*	
				Inc.FIB(AP001918)	*hlyF; ompT*	
spinach	*768	*19374597	ISEc9			*bla*_*CTX-M-15*_
			IS629		*ireA*	
			IS5		*tia*	
				Inc.FIB(AP001918)	*ompT; hlyF*	
			ISEc1		*gad*	
spinach	*784	*19374592	ISKpn8		*gad*	
***E. coli* ST162**						
spinach	*1515	*19374559		Inc.I1		*tet(A)*
				Inc.X1		*aph(3′)-IIa*
				Inc.FIB(AP001918)	*hlyF; ompT*	
			ISEc1		*(hha; yehD; yehA; yehC; yehB) (fdeC)*	
			MITEEc1		*(nlpI; terC) (terC; hra)*	
***E. coli* ST1193**						
lettuce	*1993	*19374586	MITEEc1		*yehC; yehA; yfcV; yehB; yehD*	
			ISEc31		*iha*	
			IS629		*papA_F43; sat; iutA; iucC*	
spinach	*720	*19374549	IS26			*bla*_*CTX-M-27*_
			MITEEc1		*(yfcV; irp2; yehD; yehC; yehA; yehB; fyuA; irp2) (terC)*	
			IS629		*sat; iutA; papA_F43; iucC*	
			ISEc31		*iha*	
***E. coli* ST1585**						
spinach	*788	*19374554		Inc.FIB(AP001918)	*hlyF; ompT*	
			MITEEc1		*terC*	
tomato	*790	*24818886		Inc.FIB(AP001918)	*ompT; hlyF*	
			MITEEc1		*terC*	
***E. coli* STc75778699e0a2b1faca8b5d6f9051eb7d9defca4**						
spinach	*1126	*19374556	ISEc38		*hha*	
				Inc.FII(pSE11)	*anr*	
				Inc.FIB(AP001918); Inc.FIB(K); Inc.I1		
***E. coli* ST210d7d18a802c59df81880a978149a02c49a6021**						
spinach	*1129	*19374544		Inc.FII(pSE11)	*traT; anr*	
			MITEEc1		*(yehC; yehA; yehD; yehB) (nlpI)*	
				Inc.FIB(AP001918)	*hlyF; ompT*	
			IS629		*terC; astA*	
			ISEc38		*fdeC*	
lettuce	*1131	*19374545	MITEEc1		*(yehD; yehC; yehB; yehA) (nlpI)*	
			IS629		*astA; terC*	
			ISEc38		*fdeC*	
				Inc.FIB(AP001918)	*ompT; hlyF*	
				Inc.FII(pSE11)	*anr; traT*	

## Discussion

To the authors’ knowledge, this is the first study presenting genomic information on environmental multidrug-resistant ESBL-producing *E. coli* isolates from fresh produce and irrigation water sources in South Africa. In total, 59% of the multidrug-resistant ESBL-producing *E. coli* were considered commensal based on the phylogroups. It is well known that *E. coli* is ubiquitous and forms part of the natural flora of the gastrointestinal system of humans and animals. Apart from the traditional virulence factors and toxins that define pathogenicity, molecular features such as the ability to evade the host’s immune system or a group of genes to activate other genes also contribute toward bacterial pathogenesis.^52^ In the current study, PathogenFinder was used to predict the probability of environmental *E. coli* being a human pathogen. The pipeline matched the genomic input against known pathogenic and nonpathogenic gene families as the presence of gene families containing proteins with unknown functions has also been reported to play an important role in pathogenicity,^52^ resulting in all isolates having a > 90% predicted probability of being a human pathogen. Commensal *E. coli* with no pathogenic features, as well as intestinal pathogenic strains, are most often observed in phylogroups A or B1.^54^ Correspondingly, most of the *E. coli* isolates from the current study belonged to phylogroups A and B1; however, no virulence genes associated with intestinal pathogenic strains were present.

Notably, ten *E. coli* ST58 strains belonging to phylogroup B1 were detected in the current study. However, the organisms’ ability to acquire both resistant determinants and virulence factors results in harmless commensals becoming emerging human pathogens, capable of causing a broad spectrum of intestinal and extraintestinal disease.^55,56^ Previously, *E. coli* ST58 harboring multiple antimicrobial resistance and virulence genes have been reported in store-bought fresh produce as well as from pork sausage in Germany,^57,58^ similar to the results from the current study. Although limited information is available about the ST58 serotypes detected in the current study, it is well documented that ST58 *E. coli* strains have caused human extraintestinal infections, including sepsis, and are reported as one of the main ESBL-producing *E. coli* circulating in the human–animal–environment.^59^ In food-producing environments, *E. coli* is often used to indicate fecal contamination as it appears at low background levels in the environment but has high survival rates.^60^ Furthermore, the WHO reported that ESBL-producing *E. coli* should be used as an indicator in monitoring programs to facilitate the establishment of integrated multisectoral antimicrobial resistance surveillance in One Health.^19^ Interestingly, in four isolates (two phylogroup A ST93 and two phylogroup B1 ST847 and ST58 *E. coli* isolates) combinations of *KpsMII_K5*, *iutA*, and *papC* virulence factors, among others, were present.

According to Johnson et al.,^61^ for isolates to be classified as ExPEC, two or more of the *papAH*, and/or *papC* (P-fimbriae), *sfa-focDE* (S- and F1C-fimbriae), *afa-draBC* (Dr-binding adhesins), *iutA* (aerobactin siderophore system), and *kpsMII* (group 2 capsules) virulence factors need to be present. Other strains from the current study that also harbored two or more virulence factors for the acknowledged molecular definition of ExPEC belonged to phylogroups D (from water and fresh produce samples) and B2 (from fresh produce samples). Moreover, four strains from water and fresh produce samples from the current study belonged to phylogroup G. Clermont et al.^10^ reported that phylogroup G strains are highly virulent with antibiotic-resistance potential and are closely related to phylogroup B2. These strains represent around 1% of *E. coli* in humans and, although uncommon, have previously been isolated from livestock, poultry, and poultry meat in the East of England and Northern Europe.^10^

In the current study, all phylogroup G strains belonged to the ST117 lineage, previously reported as the most prevalent lineage in phylogroup G and reported as a poultry-associated lineage with the ability to also establish in humans and cause severe extraintestinal diseases.^10^ From the current study, the phylogroup G ST117 isolates were obtained from irrigation water and fresh produce samples, and all four strains harbored the ExPEC determining virulence factors. Typically, *E. coli* strains responsible for extraintestinal infections belong to phylogroup B2, D, and F.^61,62^ The phylogroup D isolates from this study all belonged to ST69 lineages. Recently, ExPEC ST69 has been reported among the major lineages globally (“top 20 commonest ExPEC sequence types”)^63,64^ and has been isolated from raw vegetables in South Korea^65^ as well as from poultry and humans in Zambia.^66^

In contrast to previous studies, the four *E. coli* ST69 strains from the current study had different serotypes and did not harbor plasmids associated with antimicrobial resistance genes. Other common lineages among ExPEC include ST10, and in the current study, six of the phylogroup A *E. coli* isolates were characterized as the O101:H-ST10 strains. Globally, ST10 is found in different hosts, including environmental and animal sources, among others, and is considered a high-risk emerging pandemic lineage.^66−68^ Typically, serotype O101 is detected among pathogenic *E. coli*, associated with animal and human disease, with serotype O101:H9 predominantly reported in Shiga toxin-producing *E. coli* (STEC). Interestingly, the O101:H9-ST10 strains from the current study did not harbor any *stx1*, *stx2*, *eaeA*, or *ehxA* virulence genes usually associated with STEC;^5^ however, antimicrobial resistance genes from at least eight different classes were present among these strains.

Although limited studies have focused on the surveillance of nonpathogenic bacteria, the significance of commensals as reservoirs of antimicrobial resistance in the environment and food chains is gaining more attention.^15,69^ As an example, Gekenidis et al.^70^ reported on the occurrence of antibiotic-resistant environmental *E. coli* from drain water and irrigated chive plants through a complete irrigation chain with resistance determinants for up to six different antibiotic classes present. Although no clear distinction was seen between the resistance profiles of *E. coli* from irrigation water versus those of *E. coli* from fresh produce in the current study, the phylogroup E and G strains generally harbored fewer resistance genes than isolates that belonged to the other phylogenetic groups.

From the current study, 95.10% of the environmental strains showed a potential for multidrug resistance based on the genomic profile, with multidrug resistance defined as nonsusceptibility to at least one agent in three or more antimicrobial categories.^71^ This contrasts with results from a study in Uganda, where the commensal *E. coli* isolates from food animals, characterized using WGS, harbored a limited number of antimicrobial resistance genes.^15^ Notably, none of the isolates in the current study harbored the plasmid-mediated colistin resistance gene (*mcr*) or carbapenemase resistance genes (*bla*_NDM_, *bla*_KPC_, *bla*_VIM_, and *bla*_OXA-48_). This contrasts with previous similar studies in China, Brazil, Bangladesh, and Germany, where clinically relevant ESBL-producing *E. coli* harbored these genes conferring resistance to the last resort drugs for the treatment of infections isolated from water and fresh produce samples.^72−75^ However, multidrug-resistant *E. coli* isolates harboring clinically significant *bla*_CTX-M_ genetic determinants, among others, have previously been reported in water^76^ and fresh produce^70^ samples, which correspond to the results from the current study. Currently, the most prevalent ESBL globally reported in clinical isolates, human and animal fecal matter, and the aquatic environment is *bla*_CTX-M-15_.^71,77^ The predominant β-lactamase resistance genes detected in the current study were *bla*_CTX-M-14_ (CTX-M Group 9) followed by *bla*_CTX-M-15_ (CTX-M Group 1), and in selected isolates, these genes were associated with insertion sequences.

Specifically, in two isolates, *bla*_CTX-M-15_ was carried on the insertion sequence ISEc9, which corresponds to a previous study where *E. coli* was isolated from hospital patients in Nigeria.^78^ Moreover, the cocarriage of the quinolone resistance gene *qnrS1* and *bla*_CTX-M-15_ in association with insertion sequence ISKpn19 from the current study corresponds to *E. coli* characterized from dairy farms in Québec, Canada.^79^ The Inc.FII plasmid, known globally to contribute toward the spread of clinically relevant antimicrobial resistance genes,^80^ was detected in association with certain virulence and antimicrobial resistance genes in isolates from water and fresh produce samples in the current study. Within a One Health context, these results emphasize the significance of monitoring food-producing environments, including water and fresh produce, in food safety and antimicrobial resistance surveillance programs.

Mbanga et al.,^81^ reported on environmental *E. coli* from wastewater treatment plants and receiving river water in Kwazulu-Natal (South Africa) that cocarried antimicrobial resistance, heavy metal (mercury and chromate), and disinfectant (quaternary ammonium compounds) genes. In contrast, isolates from the current study did not harbor any heavy metal resistance genes. However, the biocide resistance *qacE* gene as well as the *sul1* antimicrobial resistance gene, which are typically found at the 3′ conserved segment in a class 1 integron,^82^ was present in two isolates from the current study where complete integrons were identified. Similarly, *E. coli* isolates from wastewater treatment plants in South Africa,^81^ broiler chickens in the South of Iran,^83^ and human, animal, and environmental samples from countries of the Andean Community^84^ have been reported to carry complete class 1 integrons. Integrons carrying multiple antibiotic-resistance genes or virulence genes, embedded within mobile genetic elements, significantly contribute toward bacteria across different One Health sectors acquiring traits through the cotransfer of genes, which can increase pathogenicity.^85^

It is well documented that interconnected reservoirs of antimicrobial-resistant bacteria include animals, humans, and food, which allows rapid gene exchange through horizontal gene transfer within food systems.^86^ From a food safety perspective, identifying microbial contaminants in the water-plant-food nexus is vital for hazard characterization. In African countries, including South Africa, the evidence of STEC O157:H7 occurrence in the environment and infection among animals and humans, in general, is not conclusive.^87^ Furthermore, the results from the current study correspond to previous South African studies showing a low prevalence of STEC O157:H7, usually associated with *E. coli* foodborne disease outbreaks, in fresh produce production systems.^26,30−33,87^ Although antimicrobial-resistant bacteria complicate food safety assurance,^2^ building a genomic database of the virulence genes, antimicrobial resistance genes, and potential pathogenicity of environmental isolates, comparable to existing clinical data, is essential for the implementation of risk mitigation strategies. A limitation of the current study is the use of short sequencing reads, preventing complete plasmid assembly and establishing the role of the detected plasmids in gene transfer among environmental bacteria. A recommendation for future research is therefore to combine phenotypic and long- and short-read whole genome sequencing characterization along with gene transfer studies to be able to investigate the role that plasmids play in mediating resistance within food-producing environments. To date, the genomic evaluation of antimicrobial resistance, virulence factors, and associated mobile genetic elements in nonclinical *E. coli* have not been extensively investigated.^86^ The results from the current study highlighted the important role that the environment has as a reservoir of multidrug-resistant *E. coli* and, furthermore, the critical need for continuous potential pathogen surveillance within a One Health context. Future studies should further explore surveillance of the One Health environment.

## Supporting Information Available

The assembly metrics of the whole genome sequences of *E. coli* isolated from water and fresh produce samples in South Africa (XLSX)
